# Structural, Electronic, Reactivity, and Conformational Features of 2,5,5-Trimethyl-1,3,2-diheterophosphinane-2-sulfide, and Its Derivatives: DFT, MEP, and NBO Calculations

**DOI:** 10.3390/molecules27134011

**Published:** 2022-06-22

**Authors:** Nasrin Masnabadi, Mohammad R. Thalji, Huda S. Alhasan, Zahra Mahmoodi, Alexander V. Soldatov, Gomaa A. M. Ali

**Affiliations:** 1 Department of Chemistry, Roudehen Branch, Islamic Azad University, Roudehen P.O. Box 189, Iran; 2 School of Chemical Engineering, Yeungnam University, Gyeongsan 38541, Gyeongbuk, Korea; mdthalji@gmail.com; 3 Environmental Research and Studies Center, University of Babylon, Babil 51002, Iraq; sci.huda.s@uobabylon.edu.iq; 4 Department of Chemistry, University of Applied Science and Technology, Center of Arya Gach Poldokhtar, Tehran P.O. Box 68, Iran; mahmoodi_zahra80@yahoo.com; 5 The Smart Materials Research Institute, Southern Federal University, Sladkova Str. 178/24, Rostov-on-Don 344006, Russia; soldatov@sfedu.ru; 6 Chemistry Department, Faculty of Science, Al-Azhar University, Assiut 71524, Egypt

**Keywords:** natural bond orbitals, density functional theory, density of states, stereoelectronic interactions, generalized anomeric effect

## Abstract

In this study, we used density functional theory (DFT) and natural bond orbital (NBO) analysis to determine the structural, electronic, reactivity, and conformational features of 2,5,5-trimethyl-1,3,2-di-heteroatom (X) phosphinane-2-sulfide derivatives (X = O (compound **1**), S (compound **2**), and Se (compound **3**)). We discovered that the features improve dramatically at 6-31G** and B3LYP/6-311+G** levels. The level of theory for the molecular structure was optimized first, followed by the frontier molecular orbital theory development to assess molecular stability and reactivity. Molecular orbital calculations, such as the HOMO–LUMO energy gap and the mapping of molecular electrostatic potential surfaces (MEP), were performed similarly to DFT calculations. In addition, the electrostatic potential of the molecule was used to map the electron density on a surface. In addition to revealing molecules’ size and shape distribution, this study also shows the sites on the surface where molecules are most chemically reactive.

## 1. Introduction

Chemical compounds containing organic segments that are bonded to phosphorus either directly or indirectly (e.g., via sulfur, oxygen, or nitrogen) are known as organo-phosphorus compounds (OPs) [1]. They are one of the most commonly encountered compounds and are used worldwide in a variety of agricultural [2], industrial [3], medical [4], and veterinary applications [5]. In the 19th century, the first OPs were synthesized by Lassaigne and Clermont [4]. Insecticides and chemical warfare agents were created in the 20th century by Gerhard Schrader. Chemical warfare agents, eye medications, pesticides, and lubricants are manufactured from OPs. Most researchers have recently been interested in computational methods to detect chemical species evidence [6,7,8]. Theoretical approaches are often used to study chemical processes and the molecular characteristics of compounds. Aside from that, theoretical approaches are often used to determine newly synthesized chemical species [9,10]. This work aims to thoroughly study the computational calculation for 2,5,5-trimethyl-1,3,2-dihetereophosphinane-2-sulfide, where the heteroatom is one of the O, S, or Se atoms. No theoretical studies have yet been performed on 2,5,5-trimethyl-1,3,2-dihetereophosphinane-2-sulfide and its derivatives.

The conformational equilibrium is influenced by stereoelectronic interactions, which have long been recognized [11,12]. However, Praly and Lemieux define anomeric effect (*AE*) based on a difference between equatorial (*eq.*) and axial (*ax.*) conformers’ total *endo*- and *exo*-anomeric effects [13,14]. This phenomenon is known as a “Generalized Anomeric Effect” (*GAE*) or “negative hyperconjugation” [12]. Experimental and theoretical approaches were used for the in-depth study of the AE [15,16]. Furthermore, depending on the relative amount of the distinct *GAE_eq._* and *GAE_ax._* contributions to the total *GAE*, the *GAE* associated with electron delocalization can have negative or positive values [17]. As a result, Equation (1) can compute the total *GAE* for components (**1–3**) [18,19].

(1) $$GAE=\sum {\left(GAE\right)}_{eq.}-\sum {\left(GAE\right)}_{ax.}$$

The *GAE* influences the reactivity and conformational behavior of saturated heterocyclic systems [20]. Although several literature reports argue for the primary importance of electrostatic factors, Wiberg and Rable acknowledge the complexity of the *AE* but advocate for the greater importance of bond polarization and electrostatics [21]. In addition, Mo and co-workers use Block Localized Wavefunction (BLW) analysis to disprove the hyperconjugation explanation for the *GAE* [22,23]. Then, several authors accept the existence of hyperconjugation but conclude that the electrostatic effects are more effective than hyperconjugation. Many studies on the conformations of six-membered rings, including phosphorus and sulfur, have provided convincing evidence that *GAE* can propagate through centers other than carbon atoms [24,25,26]. The more likely an electron donor is to donate to an electron acceptor, the more conjugation throughout the system. It will happen when the electron density moves from a Lewis-type NBO orbital (lone pair or bond) to an orbital that should be unoccupied (Rydberg or anti-bond). In this case, the donor–acceptor interaction keeps the electron density stable. The anomeric effect changes the conformational equilibrium around the anomeric center. It also changes the stereochemical output of several events at the anomeric center. As a result, the stereoelectronic interactions of *n*(X)^®^σ*(P-S)_app_ (P=S orientation is *ax.*) and *n*(X)^®^σ*(P-C)_app_ (P=C orientation is *eq.*) (X = O, S, and Se) were investigated. The 1,3,2-dioxaphosphorinane derivatives are interesting compounds because of their biological activities [27,28]. Numerous studies of the structures and conformations of 1,3,2-dioxaphosphorinanes and related heterocycles have been reported [29,30,31,32]. They demonstrated that anti-bonding orbitals play a role in molecules with low acceptor orbitals, such as the P=O or P=S. However, the structural, electrical, and reactive properties of these compounds, which have been examined in the literature, are limited and need more investigation.

**Scheme 1:**
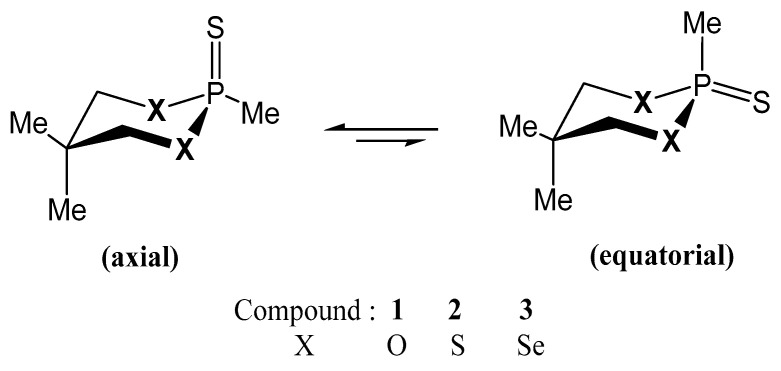
Schematic illustration between (*ax.* ⇌ *eq.*) forms of compounds **1**–**3**.

Herein, we study the structural, reactivity, and conformational (*ax.* ⇌ *eq.*) features of the 2,5,5-trimethyl-1,3,2-di-heteroatom phosphinane-2-sulfide derivatives (Scheme 1, heteroatoms = O (-oxa-), S (-thia-), and Se (-selena-)). These compounds were studied computationally using the DFT approach and natural bond orbital (NBO) analyses. These analyses were used to examine the effects of different hetero atoms in rings with different P=S orientations (*ax.* ⇌ *eq.*) and the anomeric effect in 1,3,2-dihetereo-2-sulfide (as an example of O, S, and Se-substituted analogs) on the anomeric effect. After that, compounds’ chemical reactivity and reactive site selectivity (**1**–**3**) were thoroughly investigated using DFT, molecular electrostatic potential, and density of states (DOS).

**Scheme 2:**
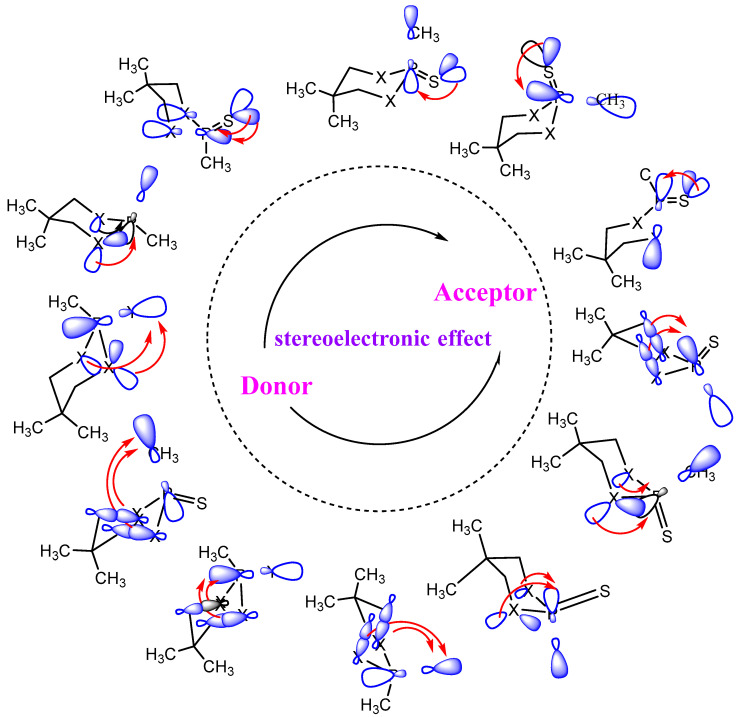
A diagram exhibiting the changes in electronic delocalization between orbitals (donor-acceptor) throughout (*ax.* *⇌ eq.*) conformations of 2,5,5-trimethyl-1,3,2-di-heteroatom (X) phosphinane-2-sulfide.

It should be known that compounds’ conformational properties affect stabilization energies. It was interesting to study the stabilization energies correlated with the electronic delocalization and the electronic structures of the model compounds (**1**–**3**) by NBO analysis [33,34,35]. The stabilization energies obtained with electronic delocalization *n*_eq_X^®^σ*P-S, *n*_ax_X^®^σ*P-S, *n*_ax_X^®^σ*P-C, σX-C6^®^σ*P-C, σX-C6^®^σ*P-S, *n*_eq_S^®^σ*P-X, and *n*_eq_S^®^σ*P-C are shown in Scheme 2.

## 2. Computational Models

The compounds (**1**–**3**) were thoroughly optimized using Gauss-View 5.0 molecular visualization software from Gaussian 03 software at the B3LYP/6-31G and B3LYP/6-311+G** levels of theory. For all computations, the DFT technique was used, which combines Becke’s hybrid gradient-corrected (three-parameter nonlocal) exchange functional [36] with Lee, Yang, and Parr’s gradient-corrected (nonlocal) correlation functional [37]. The natural bonding orbitals (NBO) 3.1 program was then used to perform an NBO analysis *ax.* ⇌ *eq.* conformations of compounds. NBO studies, including orbital population, Wiberg bond orders, and charge transfer stabilization energies at the identical B3LYP/6-31G** and B3LYP/6-311+G** levels, were conducted using the NBO 3.1 program, which is part of the Gaussian 03 software package [38].

## 3. Results and Discussion

### 3.1. Conformational Preferences

**Figure 1:**
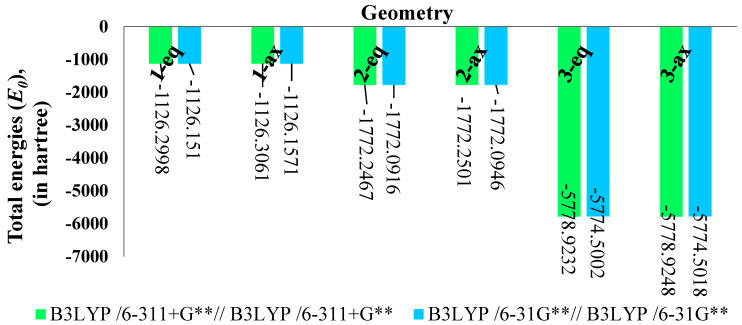
Total energies (*E*_0_, in Hartree) that are calculated for the (*ax.* *⇌* *eq.*) conformations of compounds (**1**–**3**).

**Figure 2:**
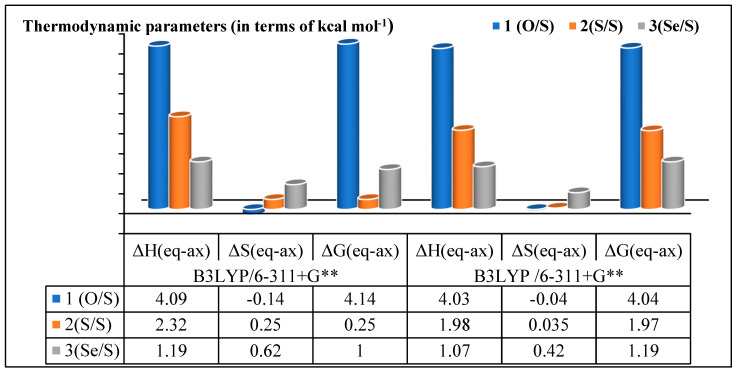
The B3LYP/6-311+G//B3LYP/6-311+G and the B3LYP /6-31G//B3LYP/6-31G calculated thermodynamic characteristics (∆*H*, ∆*S*, and ∆*G*) for the (*ax.* *⇌ eq.*) conformations of compounds **1**–**3**.

The compounds (**1**–**3**) in the chair conformations are more stable in the B3LYP approach than in the twist-boat conformations. As shown in Figure 1 and Figure 2, the total corrected energies (Δ*E*_0_, Hartree), the differences in enthalpies (Δ*H*, kcal mol^−1^), entropies (Δ*S*, cal mol^−1^ K^−1^), and Gibbs free energy (Δ*G*, kcal mol^−1^) at the standardized conditions (25 °C, 1 atm) between the *ax.* *⇌ eq.* conformations for compounds (**1**–**3**) were calculated at the levels of B3LYP/6-311+G** and B3LYP/6-31G**. Interestingly, Δ*G* values in the *ax.* conformations are lower than in *eq.* conformations. This is true for all methods used in this study, using complete geometry optimization.

**Figure 3:**
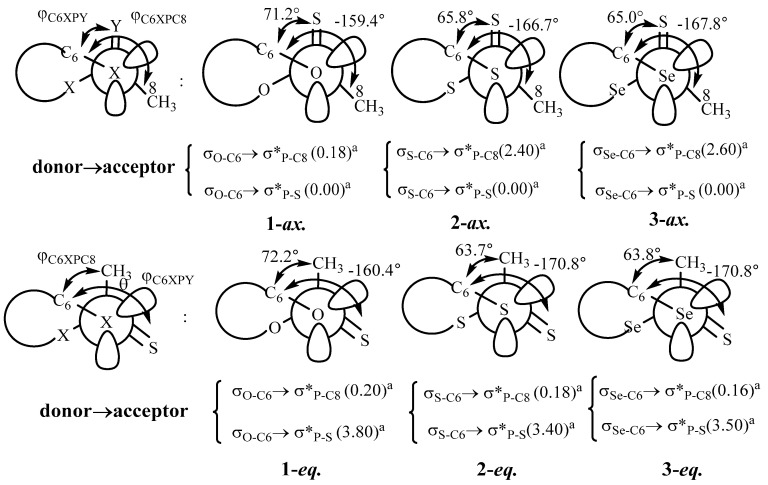
Newman projection of donor–acceptor orbitals of the compounds (**1**–**3**) in their (*ax.* ⇌ *eq.*) conformations. (^a^ stabilization energy values; kcal mol^−1^).

As shown in Figure 3, the calculated Δ*G_eq-ax_* values for compounds at the B3LYP/6-311+G level were somewhat like the Δ*G_eq-ax_* values obtained at the B3LYP/6-31G level. Δ*G_eq-ax_* values between (*ax.* *⇌* *eq.*) conformations at the B3LYP/6-311+G level of theory were found to be 4.14, 0.25, and 1.00 kcal mol^−1^ for compounds (**1**), (**2**), and (**3**), respectively. However, Δ*G_eq-ax_* values for compounds (**1**), (**2**), and (**3**) at the B3LYP/6-31G** level were calculated to be 4.04, 1.97, and 1.19 kcal mol^−1^, respectively. Notably, there is an apparent agreement between these sequences with the experimental data published in the literature for compound (**1**) [39]. Further, it is shown that the preference for *ax.* conformations compared to their equivalent *eq.* conformations decrease from compound (**1**) to compound (**3**).

Since the hyperconjugation of two electrons and orbitals occurs, stabilization is significant [40]. The effect of the molecule geometry on the energy gap, the ability of the empty orbital to receive, and the ability of the filled orbital to donate are essential parts of this scenario [41,42]. The resonance values of the energies related to *n*_ax_X^®^σ*P-Selectron delocalization are 12.48, 9.86, and 8.12 kcal mol^−1^ for the *ax.* conformations of compounds (**1**), (**2**), and (**3**), respectively. These stereoelectronic orbital interaction forms also indicate the preference of compounds (**1**–**3**) more efficiently for the *ax.* conformation. In addition, Generalized Anomeric Effects (*GAE*) values for compounds (**1**), (**2**), and (**3**) are 5.56, 3.40, and 2.90 kcal mol^−1^, respectively.

The stereoelectronic effects, which correlated to n_2X_→σ*_P-C_ electron delocalization in the *eq.* conformations and the n_2X_→σ*_P-S_ electron delocalization in the *ax.* conformations, have the most considerable degree of stability energy. The orientation of the electron’s lone pairs of the O, S, and Se atoms in the heterocyclic ring and the P-C (in *eq.* conformations) or P-S (in *ax.* conformations) for anti-bonding orbitals is antiperiplanar (*ap*) (Figure 3). Several factors influence stereoelectronic effects, such as the difference in energy between the overlapping donor–acceptor orbitals and their presence in a synclinal arrangement (*sc*) [43,44]. As a result, the σ_X-C6_→σ*_P-C8_ and σ_X-C6_→σ*_P-S_ electron delocalization, as depicted in Figure 3, cannot have high stability energy. For this reason, the stability energy values of delocalization for compounds (**1**–**3**) in the *ax.* conformations are not very important.

It is worth noting that the calculated stabilization energies associated with σ_X-C6_→σ*_P-S_ electron delocalizations for the *eq.* conformations of compounds (**1**–**3**) are 3.80, 3.40, and 3.50 kcal mol^−1^, respectively, and do not exist for the *ax.* conformations. Moreover, the electron delocalization of σ_X-C6_→σ*_P-C8_ in the *ax.* conformation increased from compound (**2**) towards compound (**3**), compared to the value of X = O, which may be due to larger amounts of s-character and electronegativity [45], and the values decreased in the *eq.* conformations from compound (**1**) to (**3**). Stereoelectronic effects are negligible in *eq.* conformations, while the stability energies of electronic transitions for *ax.* conformations are significant. According to NBO studies and thermodynamic data, stereoelectronic interactions have an important influence on compounds’ behavior.

Further, the results indicate that the values of *GAE* are more vital for analyzing the conformational preferences of these compounds than the electrostatic effects, which are less significant. Further, the results indicate that the values of *GAE* are more vital for analyzing the conformational preferences of these compounds than the electrostatic effects, which are less significant. The dipole moment of compounds was also looked at in both (*ax.* and *eq.*) orientations using the B3LYP/6-311+G computational method.

### 3.2. Bond Order and Structural Parameters

**Figure 4:**
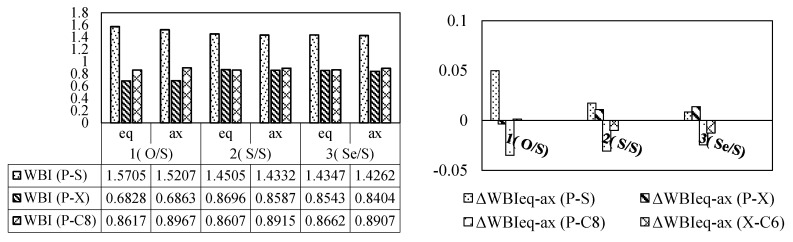
*WBI* and Δ*WBI_eq-ax_* calculation in (*ax.* ⇌ *eq.*) conformations of compounds (**1**–**3**) at B3LYP/6-311+G level.

It is possible to relate the delocalization of electrons to structural factors, such as the Wiberg Bond Index (*WBI*) term, which is the sum of squares between off-diagonal density matrix components linking atoms, using the natural atomic orbitals (NAO) [46,47]. The findings revealed that compound (**1**) has an ax’s low (nax.X→σ*P-S) electron delocalization conformations compared to compound (**3**), indicating a weakness in the covalent character [48]. In contrast, it increases the *WBI* value of P-X, whereas compound (**3**) decreases it, as shown in Figure 4. In addition, compound (**3**) has a low *WBI(_eq-ax_)* value for P=S, indicating that P=S is increasingly difficult to calculate. For the compounds examined, both the WBI(P-X) values and *GAE* agree with each other.

### 3.3. HOMO–LUMO Analysis

**Figure 5:**
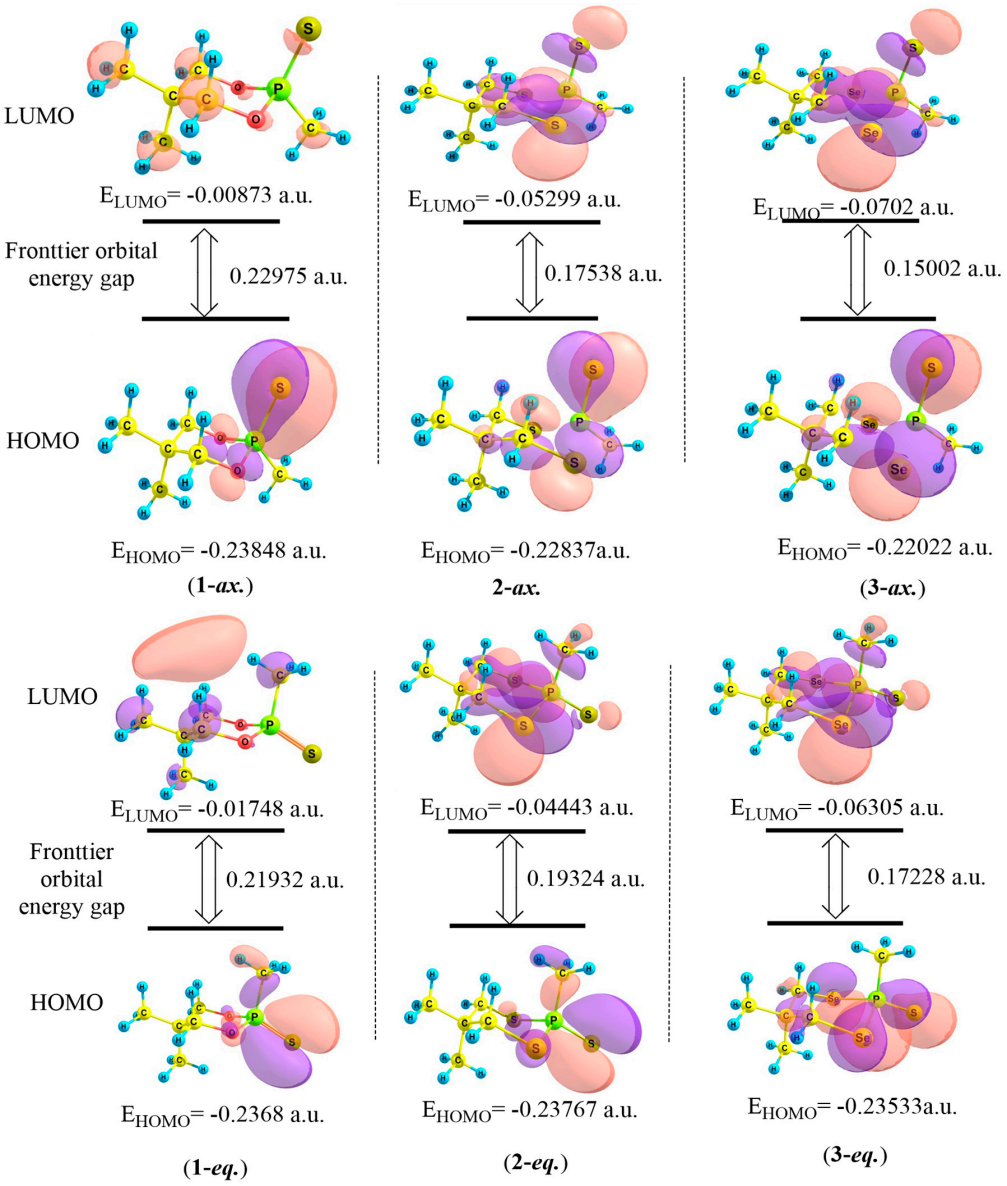
HOMO–LUMO patterns in (*ax.* ⇌ *eq.*) conformations for compounds (**1**–**3**) at B3LYP/6-311+G.

**Figure 6:**
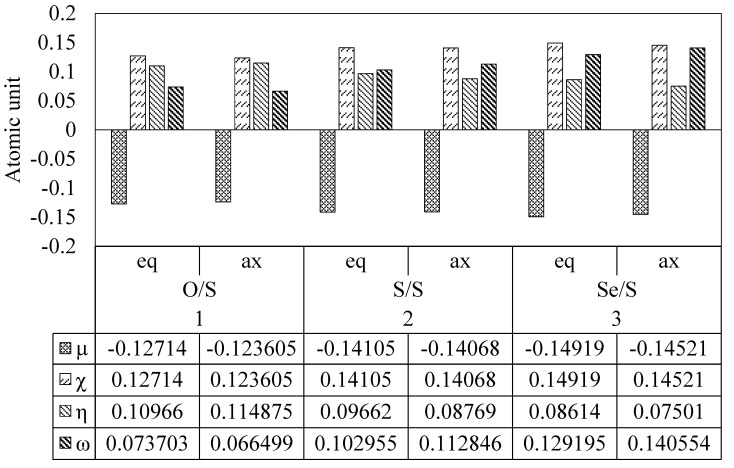
Computed quantum chemical parameters for (*ax.* ⇌ *eq.*) conformations of compounds (**1**–**3**) at the B3LYP /6-311+G level.

Both HOMO and LUMO are crucial parameters in quantum chemistry [49,50]. Typically, the ionization potential (*E_ion_*_._) is related to the HOMO energy, while LUMO energy is proportionate to an electron affinity (*E_aff._*) [51,52]. The HOMO–LUMO energy gap is derived by subtracting the HOMO and LUMO energy levels. When the energy gap is large, it suggests that the molecule has high kinetic stability and minimal chemical reactivity [53]. Furthermore, there is a relationship between the change in the binding nature of the orbitals engaged in the electronic transition and the bond distance change for each pair of bond atoms. Figure 5 depicts the energy levels of the HOMO–LUMO boundaries for compounds (**1**–**3**). It was used to measure the electronegativity (χ), chemical potential (µ), chemical hardness (*η*) and softness (*S*), global electrophilicity index (*ω*), and the measurements are presented in Figure 6. The formula for determining the calculated parameters is disclosed in Equations (2)–(5) as follows [54,55].

*χ* = −0.5 (*E*_HOMO_ + *E*_LUMO_) = − *μ* (2)

*η* = −0.5 (*E*_HOMO_ − *E*_LUMO_) (3)

*S* = 2*η*^−1^ (4)

*ω* = *μ*^2^/2*η* (5)

The purpose of the HOMO–LUMO analysis was to explore the impact of substitution (O, S, and Se) atoms on the electronic structure of the 2,5,5-Trimethyl-1,3,2-Diheterophosphinane-2-Sulfide compound. Results suggest a sensitivity of the HOMO–LUMO gap to the type of heteroatom. It was noted that the presence of O atom in the compound can reduce the HOMO–LUMO gap in *eq.* conformation. In contrast, S and Se atoms could increase the HOMO–LUMO gap in *eq.* conformation. The conclusion is that the effect of the atoms in the compound cannot be ignored when figuring out what the band gap means. Furthermore, HOMO–LUMO plots were employed to examine the compound’s hardness of chemical, chemical potential, and electro-negativity. The energy of the highest occupied molecular orbital (EHOMO) and the energy of the lowest unoccupied molecular orbital (ELUMO) are utilized in the Equations (2)–(5), based on the Koopmans approximation. These parameters are reactivity descriptors and electronic properties. A good, more reactive, the nucleophile is characterized by a lower value of μ and ω. A good electrophile is characterized by a high value of μ and ω.

The thermodynamic characteristics (∆*H*, ∆*S,* and ∆*G*) values in compound **1** are higher than in compounds **2** and **3**. Thus, the *ax.* conformation has higher reactivity than the analogous *eq.* conformation (Figure 2), and the ∆E_L-H_ value decreases as the intruding heteroatom inside the ring (O→S→Se) switches from compound (**1**) to compound (**3**) in both (*ax.* *⇌ eq.*) conformations. Depending on the η values, wide HOMO–LUMO gaps suggest a rigid molecule, while tiny gaps indicate a soft molecule, which is also more reactive than one with wide gaps. This property is crucial, for example, to understand the many types of pollutants in terms of how quickly they react and how well they attack specific sites [56]. It can be seen from Figure 6 that (*ax.* ⇌ *eq.*) conformation of compound (**1**) is almost reactive with high hardness values. Furthermore, the low *ω* values in the (*ax.* ⇌ *eq.*) conformation of compound (**1**) demonstrate electrons’ strong current from the donor moiety to the acceptor. It is important to understand that the energy gaps also reflect how charge transfer occurs within compounds, which affects biological activity, for example, and is a measure of the excitability of the molecule.

The electronegativity and hardness are used extensively to predict the chemical behavior of the compounds. A hard molecule has a large HOMO–LUMO gap and a soft molecule has a small HOMO–LUMO. The LUMO represents electron(s) accepting ability and HOMO as electron donating ability of a molecule. Therefore, the 1-*ax.* confirmation should be a better nucleophile than 2-ax. and 3-*ax.*

In comparison to other geometries, the 3*-ax.* conformation has a smaller energy gap (4.0805 eV) between the frontier molecular orbitals. This makes it easier for the charge to transfer [57]. As a result, the HOMO–LUMO bandgap has narrowed due to the electron-acceptor group’s strong electron-accepting capacity.

### 3.4. Molecular Electrostatic Potential (MEP) and Density of States (DOS) Analysis

The MEP surface is a property of electronic density that is a critical determinant of atomic and molecular possession [58]. Hence, MEP has mainly been employed to describe the chemical reactivity of several biological systems involved in electrophilic and hydrogen bonding interactions [59,60]. MEP diagram is essential for researchers looking into the physicochemical characteristics of molecules since it shows the molecule size, shape, and negative, positive, and natural electrostatic potentials [61]. It was used to figure out where nucleophilic and electrophilic attacks will most likely [62]. At each given *r′(x, y, z*) point, MEP *_V(_r′_)_* is defined as the energy of the interaction between a positive test charge (a proton) and the electric charge generated by the molecule’s electrons and nuclei, and it can be calculated via Equation (6).

(6) $$V\left(r^{\prime }\right)=\frac{Z_{\alpha }}{\left|R_{\alpha }-r\right|}-\frac{\rho \left(r^{\prime }\right)}{\left|r^{\prime }-r\right|dr^{\prime }}\;$$

where *V*(*r*′) is the electrostatic potential at *r*′, *Z_α_* is the charge on the nucleus allocated at *R_α_*, and *ρ*(*r*′) is the electron density (the first term is due to the nuclear charge and the second to the electronic distribution).

Distinct colors indicate different electrostatic potential values at the surface; red and blue regions in the MEP signify electron-rich and electron-poor regions, respectively; green represents the neutral electrostatic potential [63]. The electrophilic attack region is red (P=S group in ax. conformations of compounds **1**–**3**) in most MEP diagrams, whereas the nucleophilic attack region is blue (hydrogen atoms). Negative *V(r′)* regions are often associated with a single pair of electronegative atoms. As seen by the MEP map of the title molecule, areas with a negative potential are located over electronegative atoms (oxygen and phosphorus), whereas regions with a positive potential are located over hydrogen atoms. These locations provide information about where the chemicals may interact intermolecularly.

**Figure 7:**
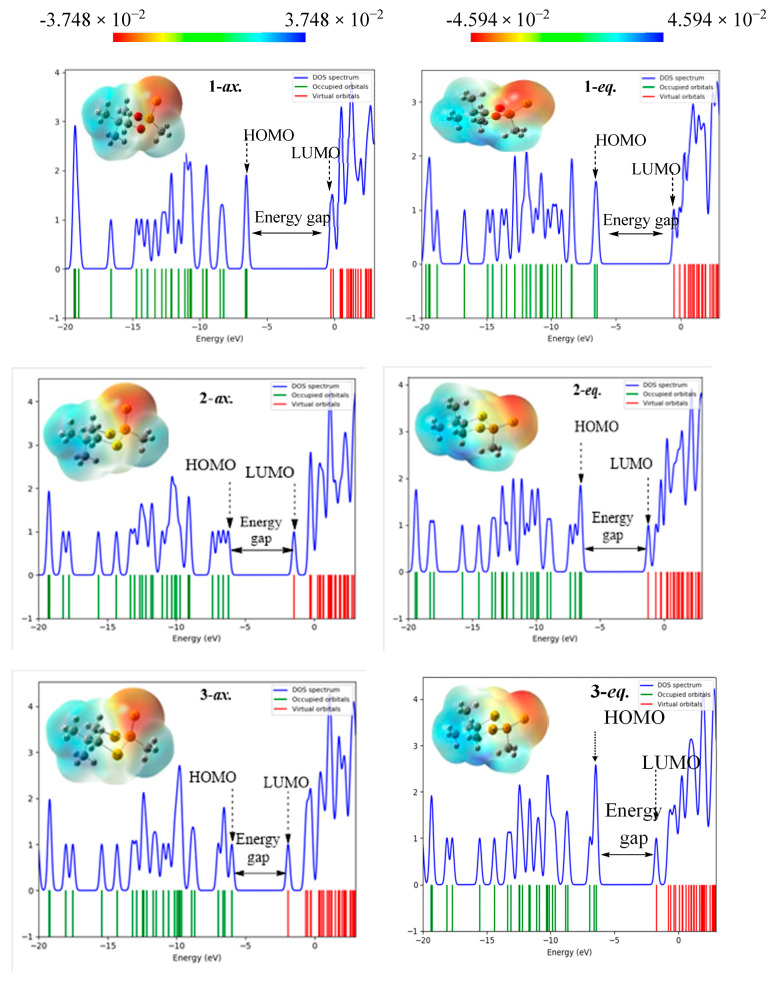
Representation of the *MEP* diagram and DOS for (*ax.* ⇌ *eq.*) conformations of compound (**1**–**3**) at B3LYP/6-311+G.

Figure 7 shows the MEP surfaces using a color code between −3.748 × 10^−2^ a.u (deepest red) to 3.748 × 10^−2^ a.u (deepest blue) for *ax.* conformations and between −4.594 × 10^−2^ a.u to 4.594 × 10^−2^ a.u for *eq.* conformations of compounds (**1**–**3**) at the B3LYP/6-311+G** level of theory. Figure 7 reveals that hydrogen atoms exhibit the greatest affinity for nucleophilic attacks, whereas oxygen atoms exhibit the greatest repulsion. In addition, the generated surface shows the molecule size, shape, and electrostatic potential value concurrently. The areas related to the (P = S)*_ax._* group in *ax.* conformations have a darker red color than the (P = S)*_eq._* group in *eq.* conformations, which means that the *ax.* conformations are more reactive than the *eq.* ones to electrophilic attack. Furthermore, the area in red in compounds **1** to **3** is decreasing. From compounds **1** to **3**, the reactivity of electrophilic reactions is declining.

The density of state (DOS, Figure 7) graph was constructed for each compound in the *ax.* and *eq.* conformations. The most common use of DOS graphs is to visualize molecular orbitals, their bonded, anti-bonded, or non-bonded nature, and their interactions [64,65]. These green and red graphs represent occupied and unoccupied regions, respectively. The energy differences between HOMO and LUMO are comparable to the values determined by DFT calculations.

Finally, DFT calculation and NBO analysis provided a clear view from a stereoelectronic perspective of the conformational priority in 2,5,5-trimethyl-1,3,2-dioxaphosphinane 2-selenide, -dithiaphosphinane 2-selenide, and -diselena phosphinane 2-selenide compounds, as reported in our recent work [66]. The findings revealed that thermodynamic properties with steric effects play a minimal role in explaining conformational behaviors of compounds, while stereoelectronic effects dominate in the justification of conformational behaviors. The anomeric effect and the electrostatic model were shown to be more successful than other effects in predicting the conformational behavior of compounds (**1**–**3**) in this study. Interestingly, conformational preference was best explained using GAE rather than electrostatic. Consequently, the GAE effects significantly control the conformational priority of compounds (**1**–**3**). Therefore, the results of this work are consistent with previously reported work. In addition, the results of DFT calculations are a good tool for predicting stereoelectronic behavior and anomeric effects, and the conformational behavior of the compounds could reasonably account for the electrostatic interactions.

## 4. Conclusions

In conclusion, the stability of 2,5,5-trimethyl-1,3,2-di-heteroatom (X) phosphinane-2-sulfide derivatives (X = O, S, and Se) corresponding to their *ax.* and *eq.* conformers was evaluated. The compounds were analyzed employing the DFT approach and the NBO interpretation. It was found that the *ax.* chair conformations are more stable than their corresponding *eq.* conformations, which could be the reason for the *ax.* conformational preferences. Furthermore, thermodynamic parameters revealed that steric effects contribute to the reasoning of compound conformational behaviors and that stereoelectronic effects are prominent in steric effects based on conformational behaviors. As a result, the enhanced stability of the relevant confirmation was ascribed to the generalized anomeric effect. The calculated *GAE* values are more important than the electrostatic effects when figuring out how these compounds like to be in a different geometry. Conformational preference was best explained using *GAE* rather than electrostatic or steric influences. Calculations were also made for the HOMO–LUMO energy gap and other molecular characteristics. Based on their interpretation of the structure–activity connection, the molecular electronic and nucleophilic centers may be deduced from the molecule’s interior center. In this way, it was shown how the parameters could help understand the compounds’ conformational and chemical properties.

## Data Availability

The data presented in this study are available in the article.
